# Management of Cervical Root Resorption on the Palatal Aspect Using Combined Internal and External Surgical Approaches: A Case Report Study

**DOI:** 10.1155/crid/5583203

**Published:** 2026-01-29

**Authors:** Sahar Shafagh, Marjan Bolbolian, Mahsa Zohdi

**Affiliations:** ^1^ Department of Endodontics, School of Dentistry, Qazvin University of Medical Sciences, Qazvin, Iran, qums.ac.ir

**Keywords:** cervical root resorption, cone-beam computed tomography, resorption treatment

## Abstract

External cervical resorption (ECR) is a progressive disease that occurs at the cervical region of the tooth, developing beneath the epithelial attachment. ECR is often asymptomatic and detected in routine radiographic examinations. This condition can significantly impact the long‐term survival of the affected tooth. Therefore, early diagnosis and proper management are both crucial and challenging. We reported a case of ECR, managed through a synchronized and simultaneous treatment approach combining surgery and root canal therapy. The defect was restored with resin‐modified glass ionomer. Nine‐month clinical and radiographic follow‐up showed satisfactory healing with no signs of recurrent resorption, and the tooth remained asymptomatic and functional.

## 1. Introduction

External cervical resorption (ECR) is an aggressive lesion at the enamel–cementum junction that destroys the tooth structure in the cervical region, which can cause tooth loss [1]. ECR has become a significant challenge in endodontics and comprehensive dental care. Its clinical manifestation is defined by an insidious, yet progressively destructive pathology [2]. After the loss of tooth structure, the resorbed area is then replaced by tissue that is highly vascular, which is characterized clinically by pinkish discoloration [3]. ECR is dynamic. When ECR is left untreated, it can increase in size and develop more root surface perforations. So, early intervention is recommended [4]. Despite its longstanding recognition, the exact etiology of ECR remains elusive, intertwined with numerous predisposing factors such as trauma, orthodontic procedures, and various surgical interventions [5].

Cone‐beam computed tomography (CBCT) is strongly recommended for accurately delineating lesion extension, evaluating the potential involvement of the pulp, and facilitating comprehensive treatment planning [6]. ECR has been further classified using CBCT, considering lesion height, circumferential spread, and proximity to the root canal. The height‐based classification includes (1) at the cemento‐enamel junction or coronal to the bone crest (supracrestal), (2) extending into the coronal third of the root and apical to the bone crest (subcrestal), (3) reaching the mid‐third of the root, and (4) progressing into the apical third of the root. Circumferential spread is categorized as A (≤ 90°), B (≤ 180°), C (≤ 270°), and D (> 270°). The proximity to the root canal is denoted by *d* (lesion confined to dentin) or *p* (probable pulpal involvement) [5]. Heithersay et al. also introduced an alternative classification system for ECR, dividing lesions based on their extent and depth of penetration. Class 1 (H1) represents a small cervical lesion with shallow dentinal involvement. Class 2 (H2) describes a well‐defined lesion extending near the pulp chamber with minimal radicular dentin involvement. Class 3 (H3) refers to a lesion that has deeply penetrated into the coronal third of the root dentin. Class 4 (H4) signifies a large lesion extending beyond the coronal third of the root. Reported treatment success rates vary by classification, with 100% success for Classes 1 and 2, 77.8% for Class 3, and 12.5% for Class 4 [7].

The selection of the most appropriate treatment for ECR is highly dependent on the progression of root resorption. Available therapeutic options include external repair, internal repair, intentional replantation, periodic monitoring, and extraction [8]. Several restorative materials have been introduced for ECR such as composite resin, amalgam, resin‐modified glass ionomer cement (RMGI), glass ionomer cement, and mineral trioxide aggregate‐based materials [9]. This study was aimed at reporting the combination of internal and external surgical treatment of a cervical root resorption that was restored with RMGI.

## 2. Case Report

**Figure 1:**
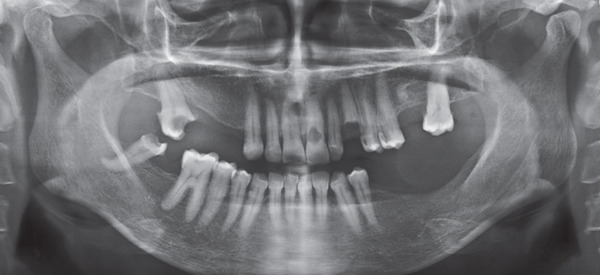
Baseline OPG.

A 57‐year‐old female patient with no significant medical history presented to the Department of Endodontics, Qazvin University of Medical Sciences in September 2025 with a chief complaint of pain in the left upper premolars. A cervical radiolucency in Tooth 9 was noticed on the orthopantomogram (OPG) (Figure 1). A periapical radiograph was taken (Figure 2). Radiographic assessment revealed a resorptive lesion with a traceable root canal outline; so, it was classified as external resorption, and internal resorption was ruled out. The tooth responded normally to thermal and electric vitality tests and was not tender to percussion. It also produced a normal percussion sound. Mobility was normal. Pink discoloration was visible at the palatal cervical region of Tooth 9 (Figure 2). The sulcus remained intact at the resorption site, with a probing depth of 2 mm around the tooth, except for 3 mm on the palatal side. CBCT was performed to evaluate the lesion’s extent, providing detailed anatomical visualization for diagnosis and treatment planning. CBCT imaging (Planmeca Promax 3D system, Helsinki, Finland) was analyzed in three planes, and the presence of cervical root resorption was confirmed (Figure 3). CBCT classification showed that the ECR in this case was classified as 2Bp (H3) [5]. Except for a slight PDL widening at the apex, no pathological change around the root apex was present. Due to the presence of a layer of predentin between the root canal and cervical resorption and normal responses to tests, we decided to proceed with surgical intervention first. If pulp exposure occurred during surgery, root canal treatment (RCT) or vital pulp therapy would be performed simultaneously.

Figure 2(A) The pinkish discoloration of the palatal aspect of the left central incisor. (B) The periapical radiograph shows a rather irregular radiolucency.

**Figure:**
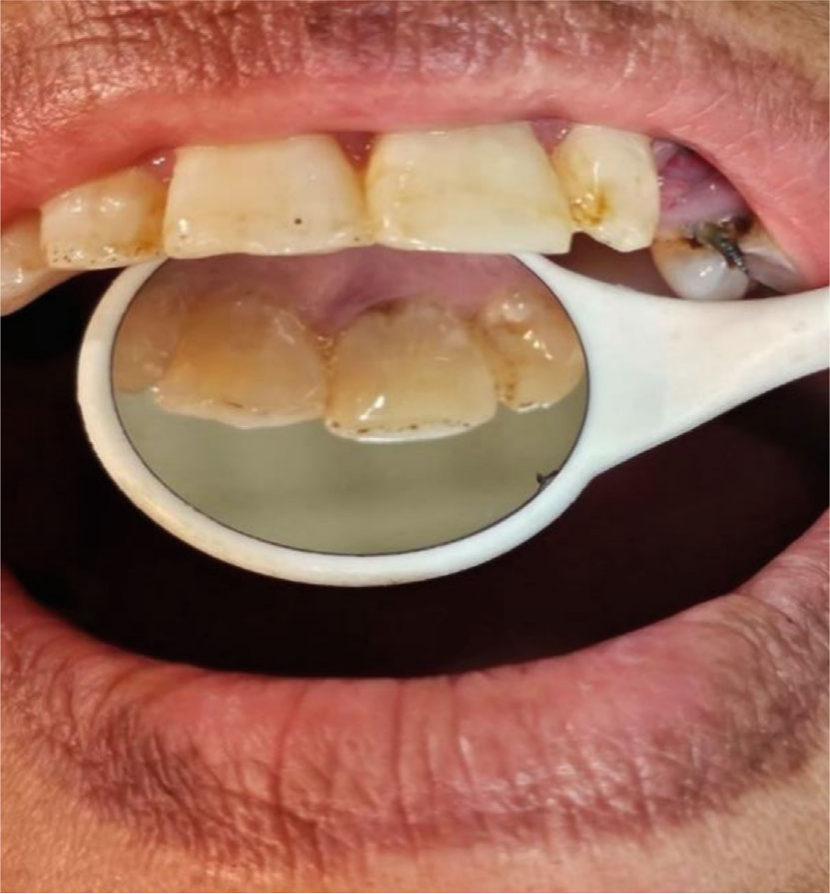
(A)

**Figure:**
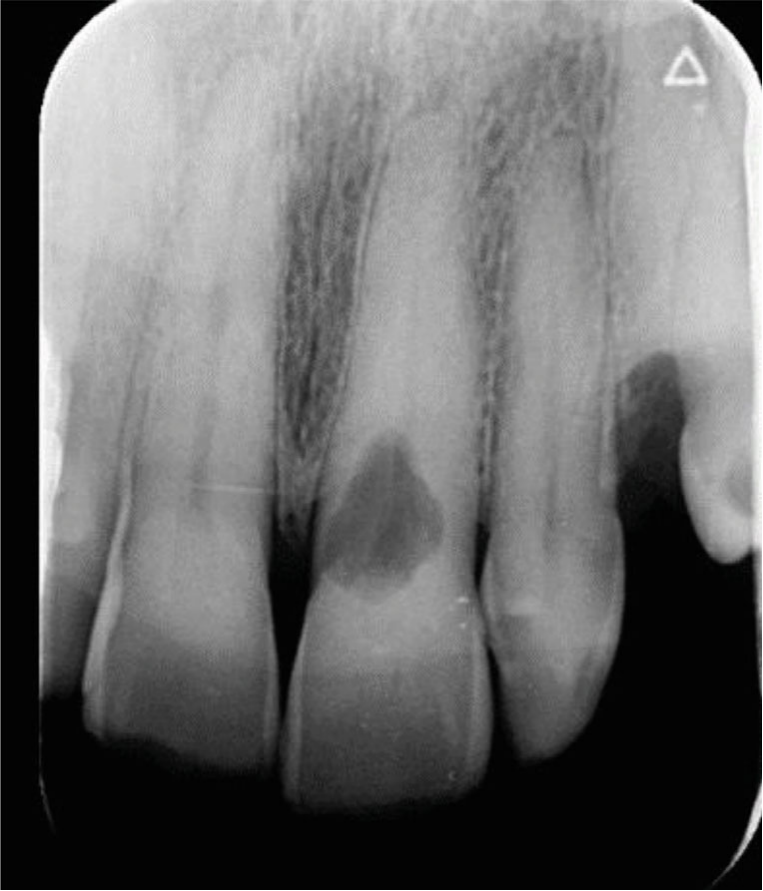
(B)

Figure 3(A) The axial plane. (B) The sagittal plane. (C) The coronal plane.

**Figure:**
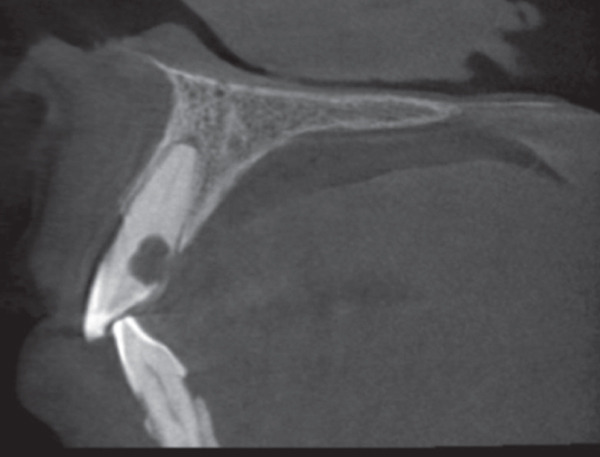
(A)

**Figure:**
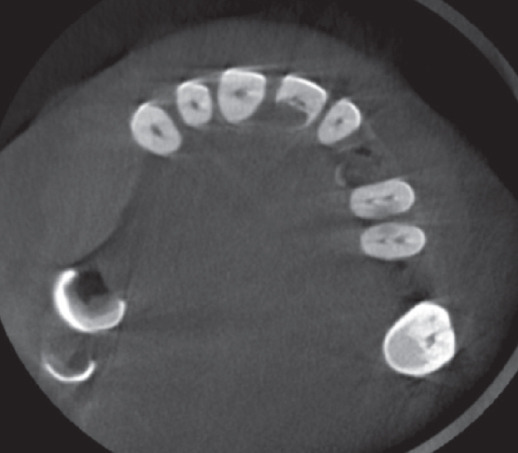
(B)

**Figure:**
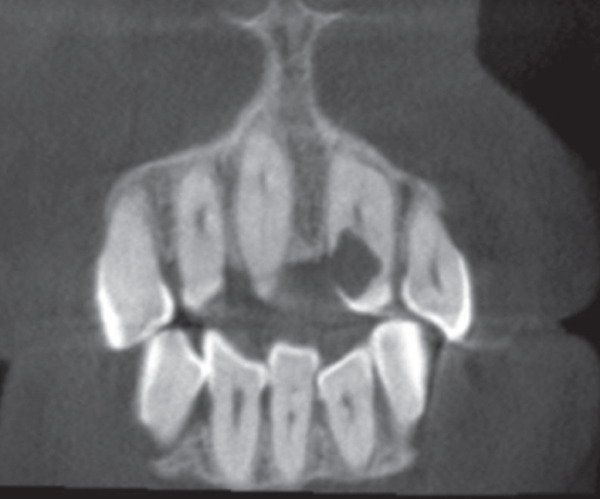
(C)

Written informed consent was obtained. The procedure commenced with preoperative antisepsis using a 0.12% chlorhexidine mouth rinse, followed by the administration of local anesthesia consisting of 2% lidocaine with 1:80,000 epinephrine (DarouPakhsh, Tehran, Iran) to achieve profound anesthesia. A palatal sulcular full‐thickness flap was raised, which allowed visualization of the resorptive tissue. Upon microscopic examination (MediWork, China), the granulation tissue over the small cervical defect present on Tooth 9 was removed with a spoon excavator, immediately placed in formalin, and sent to the laboratory for pathological examination. The resorptive cavity was debrided using a cotton pellet dipped in 90% trichloroacetic acid [10], which was applied for 1 min. Pulp exposure was identified, establishing a connection between the canal and the resorptive cavity. Based on the appearance of the pulp, which was partially necrotic, and considering the size of the exposure, it was decided to perform RCT simultaneously with the surgical procedure [11]. Isolation was achieved using a rubber dam. The working length was determined using an electric apex locator (Woodpex V, Woodpecker, China). Root canal preparation was performed using the SP1 (V‐Taper Gold, Shanghai, Fanta Dental, China) up to file F3 using the single‐length technique. Copious irrigation with normal saline was performed. At this stage of irrigation, sodium hypochlorite was not utilized to prevent damage to the soft tissue, and only normal saline was employed [12]. To avoid any obliteration of the root canal by the restorative material, a gutta‐percha cone (size 35/0.06) was placed in the canal.

We decided to restore the cavity with RMGI (Fuji II LC; GC, Tokyo, Japan). The undermined enamel was removed using a bur in a high‐speed handpiece. Hemostasis was achieved. A U‐Band matrix and wedge were established, and RMGI was placed in the cavity. Given the lesion’s location, the smoothness of the restorative material’s surface was of great importance. Therefore, finishing and polishing were performed with high precision.

After the cavity was sealed, the gingival flap was repositioned and suturing was performed using 5‐0 suture material (Supa, Iran) with the single‐interrupted technique (Figure 4). The gutta‐percha was removed. Copious irrigation was performed using 5.25% sodium hypochlorite and normal saline with a side‐vented needle, followed by passive ultrasonic activation to enhance root canal debridement.

Figure Figure4(A) A full‐thickness flap was elevated. (B) Isolated teeth with U‐Band matrix and rubber dam. (C) Gutta‐percha insertion as a barrier. (D) Restoration of cavity with RMGI. (E) Clinical appearance immediately after suturing.

**Figure:**
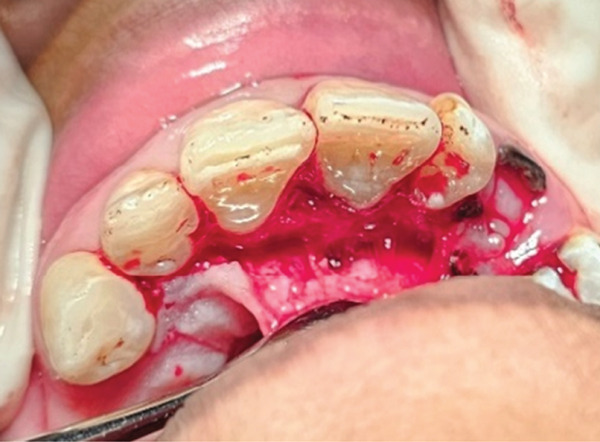
(A)

**Figure:**
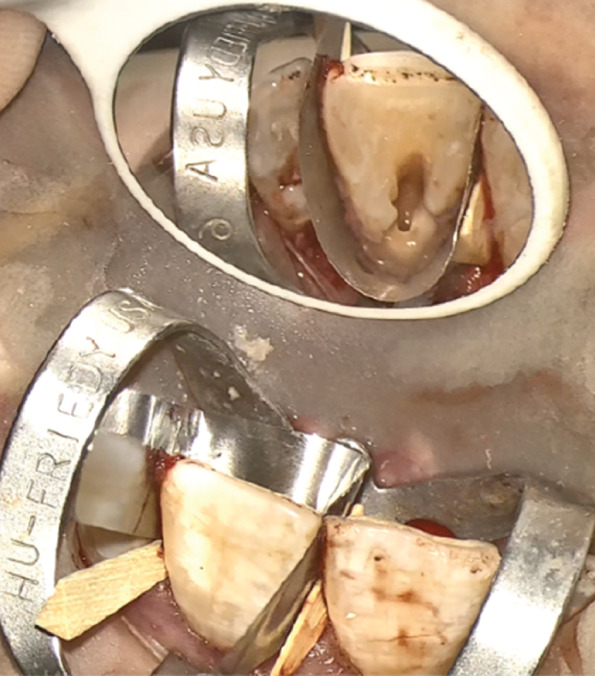
(B)

**Figure:**
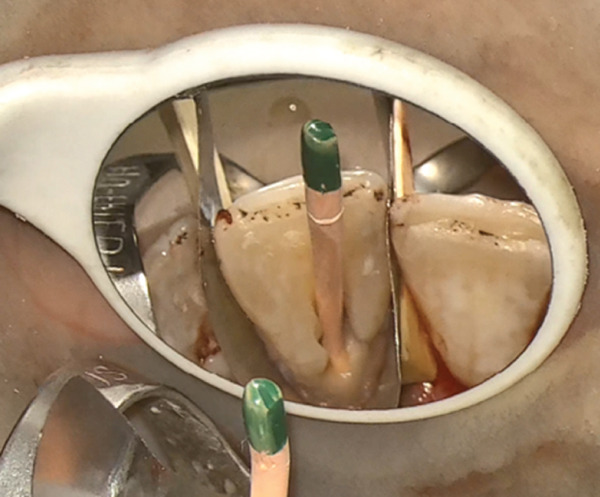
(C)

**Figure:**
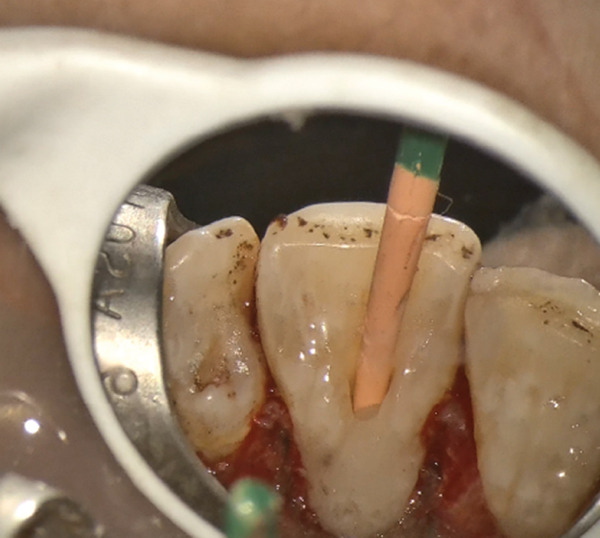
(D)

**Figure:**
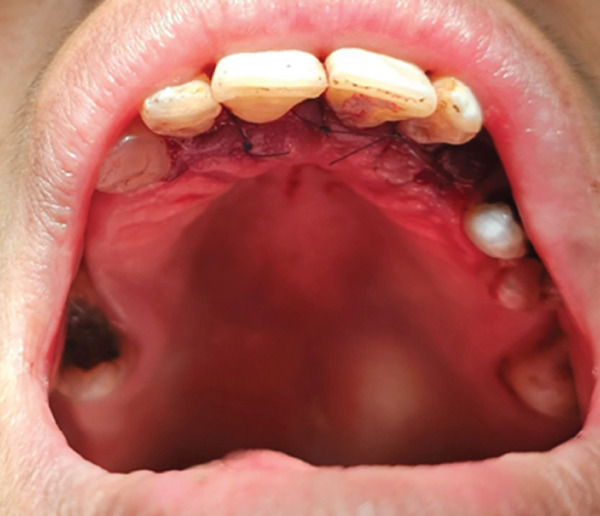
(E)

Due to the patient’s fatigue, the continuation of the RCT was postponed to the next session, and calcium hydroxide was placed in the canal. The patient was instructed on oral hygiene practices, including using a mouthwash twice daily with 0.12% chlorhexidine solution. The patient was scheduled to return in 5 days for suture removal. In the days after suture removal, RCT was performed under rubber‐dam isolation. Copious irrigation was performed using 5.25% sodium hypochlorite with a side‐vented needle, followed by passive ultrasonic activation. The smear layer was removed using 3 mL of 5.25% sodium hypochlorite, followed by 3 mL of normal saline and 3 mL of 17% ethylenediaminetetraacetic acid. The patient was advised to attend regular follow‐up visits.

The lesion was characterized by the presence of highly vascularized fibrous connective tissue within the resorptive lacunae, accompanied by inflammatory cells adjacent to the dentin surface. This suggests an active tissue remodeling process involving angiogenesis, fibroblast proliferation, and immune cell recruitment, which may contribute to pathological resorption.

**Figure 6:**
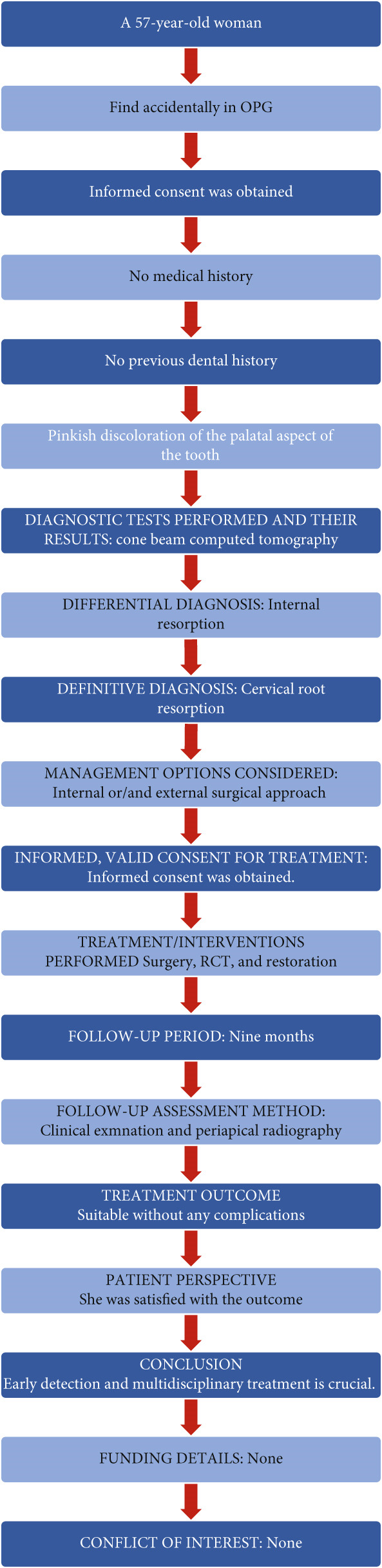
PRICE 2020 flowchart.

Follow‐up was conducted after 9 months (Figure 5). The tooth remained asymptomatic and showed normal periodontal probing depths without any recession. There was no tooth mobility. Figure 6 shows the PRICE flowchart.

Figure 5(A) Periapical radiograph immediately after the endodontic treatment. (B) Follow‐up periapical radiography after 9 months.

**Figure:**
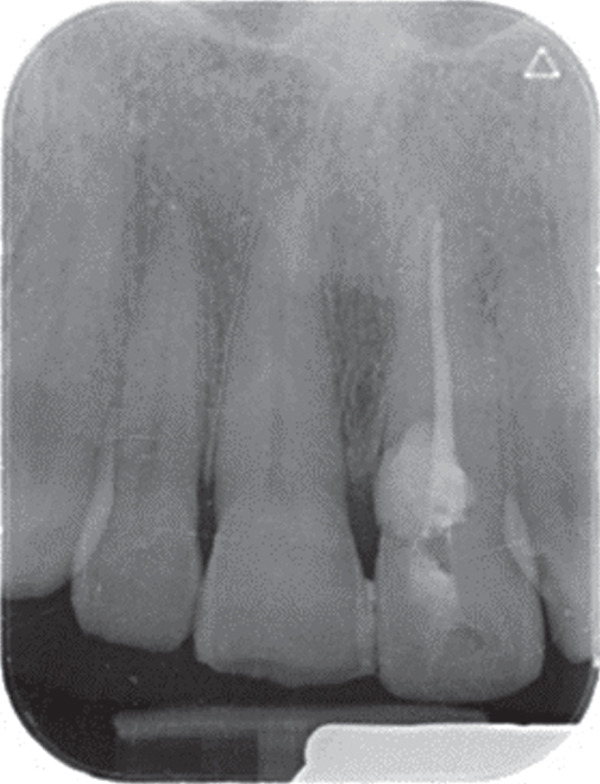
(A)

**Figure:**
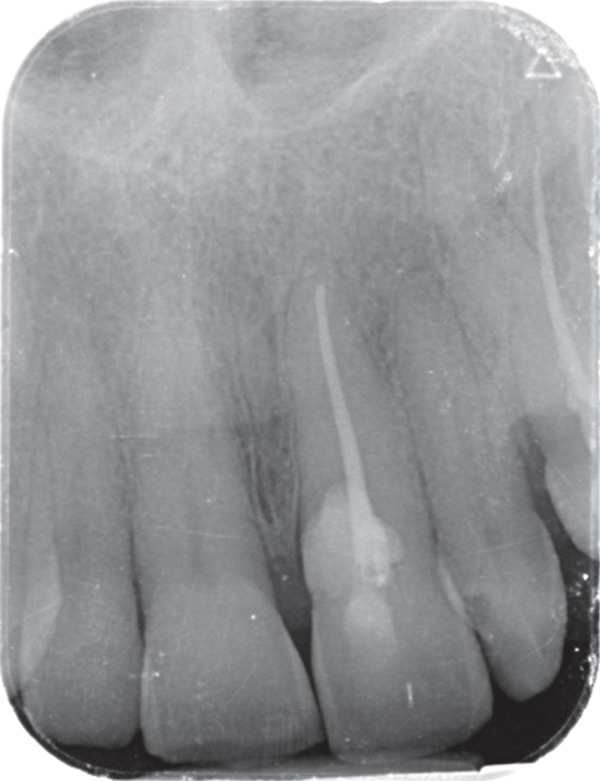
(B)

## 3. Discussion

A history of dental trauma, periodontal surgery, orthodontic treatment, Paget disease of bone, or bleaching has been proposed as a potential etiological contributor to ECR. However, in this patient, these factors were not present. It is hypothesized that the absence of posterior occlusion and traumatic occlusion of anterior teeth may serve as a plausible etiological factor contributing to the observed lesion [13].

Clinically, there is no classic presentation or particular symptomatology in the early stages of ECR, and it is commonly found serendipitously on radiographs [13]. In initial stages, vitality tests are within normal limits as long as the pulp is not involved and has not become necrotic. Internal resorption is one of the differential diagnoses of ECR [14]. Angulated radiographs can aid in distinguishing between the two [15]. Additionally, CBCT can assist in an accurate diagnosis and lead to appropriate treatment [16]. The three‐dimensional classification of ECR was proposed by Patel et al., in which height, circumferential propagation, and proximity to the root canal are analyzed. According to this classification, in the current case, the lesion was classified as 2Bp [5]. CBCT images suggested a probable thin, intact predentin layer surrounding the pulp. Also, the tooth responded to pulp sensitivity tests within normal limits, with slight PDL widening in the apex, which could be a sign of traumatic occlusion. Therefore, we decided to proceed with surgical treatment first, and if pulp exposure occurred, RCT would be performed simultaneously [11, 17]. One of the critical aspects of this technique is to ensure that the root canal does not become obliterated with RMGI. Therefore, a gutta‐percha cone was placed in the canal to serve as a protective barrier against the RMGI.

Trichloroacetic acid facilitates better removal of resorptive tissue in areas with limited access. Proper isolation is essential due to its potential adverse effects on adjacent tissues [18]. In this case, rubber dam isolation was employed to minimize unintended exposure and to aid moisture control during RMGI restoration.

In the literature, repair materials such as mineral trioxide aggregate, Biodentine, amalgam, composite resin, glass ionomer cement, and RMGIC have been used for the treatment of resorption, and the formation of periodontal reattachment is possible with these materials [19]. RMGIC is recommended for sealing supracrestal resorption areas that are in contact with oral fluids to prevent washout of the restoration [20–23]. Also, it has been advocated as a restorative material of choice due to its desirable properties, for example, biocompatibility, fluoride release, and chemical adhesion to dentin [24] Additionally, RMGI is appropriate for the esthetic zone and prevents future discoloration of the tooth [20].

Bioactive materials such as mineral trioxide aggregate and Biodentine are used in cases where the pulp is vital and a small exposure is present, or in cases with subcrestal resorptions [25–27]. In some cases, a sandwich technique was used, and mineral trioxide aggregate was covered with RMGI [11, 28]. The use of bone graft materials and membranes for guided tissue regeneration is recommended; however, there is a possibility of adverse effects on some restorative materials [29]. In this case, due to adequate access and the lack of need for bone removal, bone graft material was not used.

## 4. Conclusion

Early diagnosis and a multidisciplinary treatment strategy in the management of ECR is crucial. This case report highlights a combined internal and external surgical approach for ECR. Accurate diagnosis, careful planning, and proper sealing of the defect contributed to a successful clinical and radiographic outcome.

## Consent

Written informed consent was obtained from the patient to publish this report in accordance with the journal’s patient consent policy.

## Disclosure

All authors read and approved the final manuscript.

## Conflicts of Interest

The authors declare no conflicts of interest.

## Author Contributions

All authors had contribution to the drafting and scientific revision of the manuscript.

## Funding

The authors received no specific funding for this work.

## Data Availability

The data that support the findings of this study are available on request from the corresponding author. The data are not publicly available due to privacy or ethical restrictions.
